# The effects of decentralising antiretroviral therapy care delivery on health outcomes for adolescents and young adults in low- and middle-income countries: a systematic review

**DOI:** 10.1080/16549716.2019.1668596

**Published:** 2019-09-27

**Authors:** Roxanna Haghighat, Janina Steinert, Lucie Cluver

**Affiliations:** a Department of Social Policy & Intervention, University of Oxford, Oxford, UK; b Department of Psychiatry and Mental Health, University of Cape Town, Rondebosch, South Africa

**Keywords:** HIV, antiretroviral therapy, decentralisation, youth, healthcare, facility-based, adolescents

## Abstract

**Background**: Decentralisation of antiretroviral therapy has been implemented to scale up HIV care provision for patients in resource-limited countries. Youth living with HIV demonstrate the poorest care outcomes, compared to other age groups.

**Objectives**: To systematically evaluate evidence on the effects of decentralising facility-based HIV care on care outcomes for youth living with HIV in low- and middle-income countries.

**Methods**: A systematic review was conducted through 12 electronic databases of peer-reviewed articles, conference abstracts, and grey literature; contacting relevant experts; and hand-searching references. Records were included if they were published after 1 January 1996 (advent of triple-drug ART) and reported health outcomes for decentralised and centralised care, separately, or evaluated the effect of decentralised care on care outcomes. Two authors independently screened search results. When age-disaggregated data (10–24 years old) were required for inclusion, we contacted study authors for data abstraction. Implementation fidelity of decentralisation, study quality, and risk of bias was assessed using the TIDieR checklist, CASP checklists, and ROBINS-I tool, respectively.

**Results**: Of 11 potentially eligible studies, two studies from sub-Saharan Africa met inclusion criteria after data disaggregation by age. The studies and abstracted data were insufficiently homogenous in implementation and study design to justify meta-analysis. However, evidence suggests the potential for decentralised care to result in at least equivalent attrition-related outcomes (retention in care and mortality) for youth within decentralised HIV care. Limited sample size and significant selection and allocation bias confound clear, generalisable conclusions for youth living with HIV in resource-limited settings.

**Conclusions**: There is a paucity of evidence for the effects of decentralising HIV care for youth living in resource-limited settings, particularly recent evidence reflective of the current HIV care landscape. Further work is required to rigorously analyse the effects of decentralising HIV care to inform policymakers and care providers, particularly as demand for HIV care in this population grows.

## Background

The expansion of access to life-saving antiretroviral therapy (ART) has averted the deaths of over 7.6 million people since the mid-1990s, including nearly 5 million in sub-Saharan Africa [1]. In the third decade of the HIV/AIDS epidemic, the disease burden has shifted to low- and middle-income countries (LMICs), which currently house 90% of the world’s HIV-infected population [2,3]. The efficacy of ART has also turned HIV infection into a manageable, long-term illness, such that perinatally infected children are able to survive through to adolescence and adulthood [4].

Adolescents and youth represent 37% of new global infections and have consequently been described as the ‘centre of the epidemic’ [5–7]. As children survive to adolescence and youth, they experience increasing ownership of their health and medicine-taking practices, in a crucial transition phase. Yet, compared to other age groups, adolescents and youths living with HIV demonstrate the lowest rates of ART adherence, poorest health outcomes under care, and lowest access to and utilisation of health-care services [8,9]. Adolescents are the only age group for which AIDS-related deaths are not decreasing [10,11]. AIDS remains the leading cause of death among adolescents in Africa, and the number of AIDS-related deaths has tripled since 2000 [9].

Even when enrolled in care, HIV-positive adolescents in sub-Saharan Africa demonstrate particularly poor health outcomes, as compared to adults [12–16]. In a cohort study using routinely collected data from 160 HIV clinics in Kenya, Mozambique, Tanzania, and Rwanda, youths aged 15–24 had the highest attrition in care rates (31.1%) compared to all other age groups, including early adolescents and adults [17]. Longitudinal cohort studies have confirmed these findings: a study using data from 1999 to 2006 in southern Africa reported lower adherence rates for adolescents compared to adults after 12 months on ART (14.3% vs. 27.6%) [15]. Similarly, a study from rural Uganda reported that only 65% of adolescents on ART were retained in care from 2006 to 2011 [18]. Further, longitudinal studies in South Africa and southern Africa have reported that adolescents (11–19 years old) are less likely to achieve viral suppression and more likely to experience shorter time to viral rebound, compared to adults [13,15].

In LMICs, improved survival of HIV-positive children, combined with high new HIV infection rates among youth, has created a large, growing population of youth requiring ART [3]. In parallel, UN guidelines have called for massive efforts to scale up ART initiation and treatment maintenance globally. Guidelines also call for ART initiation at increasingly earlier stages of infection, such as the enrolment of all HIV-infected persons on ART by 2030 [9,19]. To achieve such levels of scaled-up care with limited resources, LMICs began decentralising HIV care in the mid-to-late 2000s, in alignment with recommendations from the World Health Organisation [20–22].

The primary goals of decentralisation are to expand healthcare accessibility and availability by shifting the majority of care from centralised hospitals to primary care clinics [23]. Thereby, decentralisation increases the number of facilities and health-care professionals within them that can provide basic care within them – such as monthly antiretroviral provision [24,25]. Additionally, decentralisation brings care closer to patients for whom hospitals in urban centres may be inaccessible. Although decentralisation theoretically allows for a greater number of people to access HIV care, it also requires patients to engage with a new type of healthcare – including different facilities with fewer resources and different care providers, generally with lower levels of training.

Decentralisation of HIV care is a mode of differentiating HIV care, wherein stable patients are down-referred to lower-level health-care facilities such as community health centres or primary care clinics [26]. Alongside the scale-up of ART initiation, WHO guidelines have emphasized the need to scale-up differentiated service delivery [27]. Hence, decentralising facility-based HIV care is both a form of differentiating care, and further differentiated care can be delivered within decentralised care, considering the particular care needs for specific patient populations [28]. For instance, in South Africa, even patients adopting further decentralised adherence strategies such as central chronic medicine dispensing and distribution (CMDD) programmes must engage with facility-based decentralised care through regular clinical appointments for examinations and blood tests at primary care clinics [28].

One systematic review of decentralising HIV treatment among adult and paediatric populations in LMICs suggested the non-inferiority of this care delivery model [26]. Similarly, two systematic reviews reported at least comparable outcomes when comparing routine care to task-shifted ART delivery for adults and children from physicians to non-physician health-care workers in sub-Saharan Africa [29,30]. Task-shifting care is often a key component of decentralisation, but these two interventions can be implemented separately or together. However, none of these reviews specifically examined outcomes among adolescents or youth as a distinct category, so their findings may not apply to young people.

To date, there have been no systematic reviews published in English that evaluate the effects of facility-based decentralisation of ART care among HIV-positive adolescents and youths in LMICs. This gap in evidence is particularly concerning given that facility-based decentralisation is currently being scaled up throughout LMICs as a strategy for improving ART coverage and maintenance. Evidence on the efficacy of this mode of delivery for youths living with HIV is required to understand how best to optimise care delivery and outcomes. The importance of this research is further underscored by the fact that HIV-positive youth continue to demonstrate the greatest difficulty in accessing and maintaining HIV care.

Therefore, it is imperative to evaluate the effects of decentralising HIV care on health outcomes for adolescents and youths living with HIV in LMICs, compared to those receiving centralised care. This review systematically assesses evidence for the effectiveness of decentralising ART care delivery on health outcomes of HIV-positive adolescents and youth in LMICs.

## Methods

We conducted a systematic review of the evidence following Cochrane Collaboration guidelines, according to a registered protocol (PROSPERO Registration #CRD42016051907) [31].

### Inclusion criteria

Inclusion criteria for the review were the following:

- Comparative study design (pre-/post- or multi-arm comparison groups) evaluating the decentralisation of ART delivery (ART initiation, follow-up care, or both) from centralised facilities to lower-level health centres and clinics, compared to routine care in a centralised facility (i.e. hospital) at the same time and province as the intervention arm.
- Study population includes HIV-positive adolescents and youth (10–24 years old) who were initiating or already enrolled on ART in an LMIC. Adolescents are defined as 10–19 years old and youths as 15–24 years old by the World Health Organisation [32].
- Measures health-related outcomes: mortality, loss to follow-up, attrition from care, ART adherence, viral load, CD4 count, WHO disease stage, morbidity, or changes to second- or third-line ART regimens.

Interventions comparing facility-based to home-based care were not included within this review, as this review focuses on decentralisation within facility-based care. Studies that evaluated task-shifting of care from physicians to non-medical practitioners or lower-level health practitioners, such as nurses, were not included if they did not also evaluate the decentralisation of care across facility levels.

### Search strategy

We searched 12 electronic databases and conference archives for publications within the period of 1 January 1996 (the advent of triple-drug ART) to the initial date of search (11 October 2016). We searched from 1 January 1996 because 1996 marked the advent of triple-drug ART, which has been the standard of care for HIV since its development. In a subsequent search, we updated all 12 searches for publications between October 2016 and the new search date (15 February 2019). The 12 databases, which include grey literature, were as follows: PubMed, EMBASE (1996-present), Cochrane Central Register of Controlled Trials, CINAHL, Web of Science, WHO African Index Medicus, OpenGrey, Grey Literature Report, Clinicaltrials.gov, WHO International Clinical Trials Registry Platform, International AIDS Conference abstract archive (2001–2017), and Conference on Retroviruses and Opportunistic Infections (CROI) abstract archive (2014–2017). Searches were limited to the English language, and terms were entered only in English. A sample of the search strategies used for these databases is provided in Additional File 1.

Further, we contacted selected experts in the field, including staff members of relevant international organisations and leading researchers in the field, in order to identify additional completed or on-going studies as well as any unpublished or internal reports. The following key journals were hand-searched for relevant articles in the original and updated searches: *Journal of Acquired Immune Deficiency Syndromes* (1996–2018), *The Lancet* (1996–2018), *The Lancet HIV* (2014–2018), *PLoS One* (2006–2018), *Journal of the International AIDS Society* (2000–2018), *Current Opinion in HIV and AIDS* (2006–2018), and *AIDS and Behaviour* (1997–2018). Reference lists of all studies selected for full-text screening were reviewed for additional relevant studies.

### Screening

Two reviewers (RH and JS) independently reviewed the records identified by the search strategy to determine inclusion. Full-text articles of selected abstracts were examined by both reviewers for final determination of potential study inclusion. Disagreements about inclusion were resolved by consensus.

### Data extraction and analysis

One reviewer (RH) abstracted data using a standardised data extraction form developed in consultation with the Effective Practice and Organisation of Care (EPOC) data collection form. Extracted study information included the following: study author, location, year, study and analytic designs, patient population, sample size, follow-up period, type of ART care, intervention details, comparison groups, and outcomes [33]. When studies reported at least 50 youth living with HIV in their sample but age-disaggregated data were not available within the publication, authors were contacted for age-disaggregated data. If age-disaggregated data were provided, studies were included in the review.

For non-randomised controlled trials, quality of evidence was assessed using the Critical Appraisal Skills Programme (CASP) Checklist [34]. To determine implementation fidelity, the Template for Intervention Description and Replication (TIDieR) checklist was completed and compared between included studies [35]. Risk of bias for non-randomised studies was assessed using the Risk of Bias in Non-randomised Studies of Interventions (ROBINS-I) Assessment Tool [36].

Meta-analysis was deemed inappropriate due to the small number of included studies, significant differences in intervention implementation across included studies, and low quality of evidence. Instead, we present a descriptive summary of findings from included studies.

## Results

**Figure 1.**
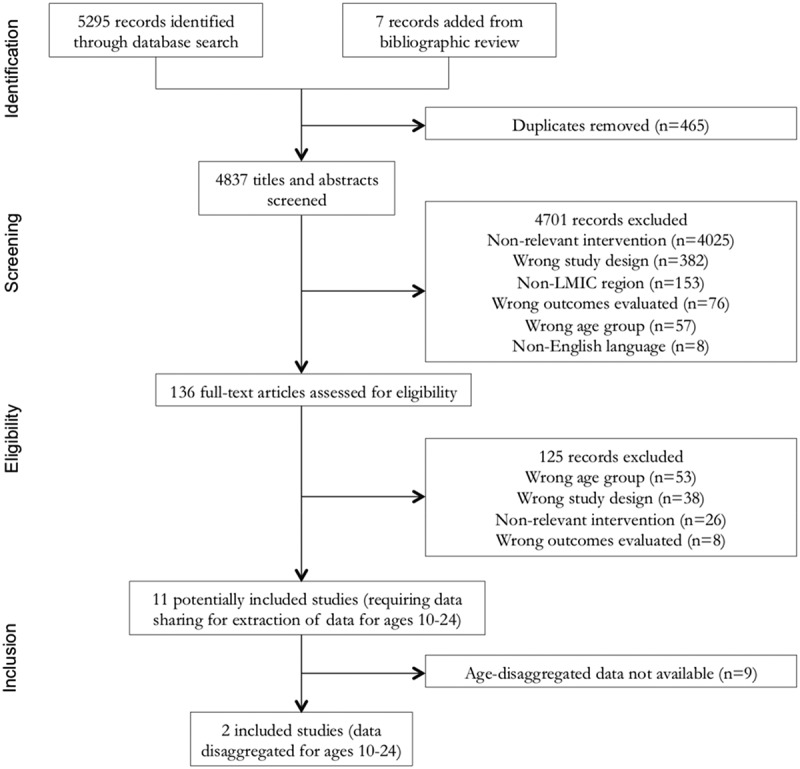
PRISMA flowchart of studies screened and selected for systematic review. LMIC: low- and middle-income country.

The systematic literature search yielded 5295 records, including grey literature, and 7 records were added from bibliographic review of key articles (Figure 1). After an initial removal of duplicates, 4701 records were excluded by screening titles and/or abstracts. Full-text articles of 136 publications were reviewed to determine eligibility for inclusion. At this stage, 125 records were excluded, the majority of which were studies in the wrong age group. We identified 11 studies that were potentially eligible. All 11 studies’ study samples were not limited to youths and required data to be age-disaggregated for inclusion in the review [22,37–46]. The lead author contacted study authors for all 11 studies at the end of each record screening round, and only studies whose authors provided age-disaggregated data were included in the review.

Ultimately, we identified two studies that met the inclusion criteria because age-disaggregated data for adolescents and youth (10–24 years old) were available [37,38].

### Study descriptions

Table 1 provides a descriptive overview of the included studies. Both studies were conducted among mixed paediatric and adult populations residing in rural sub-Saharan Africa, one in Uganda [37] and one in Malawi [38]. Within this review, study outcomes and sample characteristics are described only for adolescent and youth participants aged 10–24 at baseline in the study.

**Table 1.** Summary of included studies, listed in reverse chronological order.

Study author(s) and year of publication	Study design	Study location and dates	Total participant population and sample size	Setting	Mode of decentralisation and visit frequency	Cadres of healthcare providers at decentralised sites	Criteria for decentralisation eligibility	Outcomes measured
Scheibe et al., 2013 [37]	Retrospective cohort study	Iganga District, Uganda, April 2004-September 2009	All patients who were diagnosed with HIV and registered on ART in the included facilities during study period, n = 973 (decentralised n = 271) Gender: 63.0% female, 37.0% male Age (median, IQR): 36 (30–43)	1 general hospital and 4 health centres (3 level IV, 1 level III)	ART management (from December 2005) and initiation (from February 2007) Visit frequency: monthly	Nurse, clinical officer, midwife	N/A	Attrition (not attending facility for >90 days before audit, excl. transfers) Effective coverage (ratio of number of patients currently receiving ART: number of people living with HIV needing ART) Probability of retention in care
Chan et al., 2010 [32]	Retrospective cohort study	Zomba District, Malawi, October 2004-December 2008	All patients who initiated ART in the included facilities during study period, n = 8093 (decentralised n = 3440) Gender: 63.0% female, 37.0% male Age (mean, SD): 33.2 (±13.6) in centralised, 35.0 (±16.7) in decentralised	1 tertiary hospital and 16 rural health centres	ART management (from March 2007) and initiation (from April 2008) Visit frequency: monthly for first 6 months after initiation, then as per provider assessment	Physician (visiting), clinical officer, nurse, medical assistant	National criteria: 1. Stable on first-line ART for >3 months 2. No active opportunistic infection or drug intolerance 3. ART provider confidence in patient adherence 4. Patient closer to health centre than hospital	All-cause mortality Defaulting (not seen for >3 months after scheduled follow-up)

IQR: Interquartile range; N/A: Not available; SD: Standard deviation.

Both studies were designed as retrospective cohort analyses of decentralising ART initiation and management, with task-shifting of care integrated into both interventions. Significant differences in the implementation of decentralisation – and any co-interventions – are further discussed below. Both studies recruited participants using registration records from included facilities, including all HIV-positive patients enrolled in care from facility inception. Analysed data were comprised of clinical records extracted from patient records and HIV registers. Data coverage for both studies began in 2004, but collection ended in December 2008 for the Malawian study [38] and September 2009 for the Ugandan study [37]. For adolescents and youths, available outcomes for analysis were attrition in care (including mortality) for the Ugandan study [37] and retention in care and mortality, separately, for the study in Malawi [32].

In the Iganga District of eastern Uganda, decentralisation of ART maintenance began by shifting care from the public general hospital in town to four rural health centres between December 2005 and May 2007 [37]. In February 2007, both ART initiation and management were decentralised to these health centres, with provision of the necessary physical resources and care provider training. These health centres were open once or twice a week and outfitted to provide HIV-positive patients with 30-day supplies of ARVs. Health centres also provided HIV testing, one ART register per facility, and equipment for routinely weighing patients, although no CD4 counting equipment was available in the district. Drug supplies at health centres were delivered by the National Medical Store under the Ugandan Ministry of Health. At the health centres, care was provided by clinical officers, nurses, or midwives who had undergone the standardised national training course for comprehensive HIV care and ART provision. Study authors evaluated clinical records retrospectively in an observational study, without involvement in the delivery of decentralised care [37].

In southern Malawi’s Zomba District, the decentralisation and scale-up of ART care was facilitated through the Malawian Department of HIV and AIDS, with assistance from Dignitas International, a Canadian humanitarian NGO [32]. In the district, decentralised ART management beyond the single tertiary hospital began in March 2007. Decentralisation of ART initiation began in April 2008. At the 16 decentralised rural health centres in the study, ART care was delivered with the co-intervention of an integrated primary care model unique within Malawi, such that HIV services were integrated into routine outpatient services. To facilitate both scale-up and integrated care, Dignitas International provided intensive physical and human resources, including staffing at facilities as well as biweekly supervision, monthly mentorship, and training support for lower-cadre Ministry of Health healthcare workers. Across the 16 decentralised health centre sites, care was provided by 2 physicians visiting on a monthly basis, 5 clinical officers, 20 medical assistants, 70 nurses, 16 ART clerks, and 16 ART counsellors [32]. Further details about the implementation of decentralisation in the two studies, including the breakdown of staffing across the 16 facilities, are provided in Additional File 2.

Overall, considerable heterogeneity was observed in the design and implementation of decentralisation between the two studies. Notably, the study in Malawi delivered decentralised care alongside an integrated primary care model with intensive support from Dignitas International. Furthermore, age-disaggregated data from both studies were significantly limited in scope and depth. Age-disaggregated data for the Uganda study only provided comparative findings on one of the three outcomes in the full study – attrition. Age-disaggregated data provided for the Malawi study were only uncontrolled summary data for outcomes in the two study arms. Without individual-level data for covariates, adjusted analyses or meta-analysis between studies was not possible.

### Assessment of study quality and risk of bias

Following assessments via checklist for cohort studies, both studies were judged to have fair methodological quality. The study in Malawi by Chan et al. had a large sample of adolescents and youths aged 10–24 that allowed for precise estimates (n = 1062) [32]. However, the study in Uganda by Scheibe et al. had limited data available for the same age group (n = 56), resulting in low precision of youth-specific outcomes [37]. The two cohort studies provided limited controls for baseline differences between the decentralised and centralised patient arms – such as baseline viral load or CD4 count, which would have reflected differences in immunovirological function at the study onset. Chan et al. adjusted analyses only using WHO stage at ART initiation [32]. Scheibe et al. controlled analyses for most recent WHO stage, which most likely reflects a morbidity outcome, rather than a baseline characteristic for adjustment [37]. Additionally, the latter study applied complete-case analysis, excluding 16 youth patients missing any form of data, and potentially significant differences from included participants were not evaluated [37].

Finally, through using only retrospective review of clinical records to identify care-terminating outcomes, both studies face significant threats to the validity of measurements of mortality and, consequently, attrition from care. Previous studies in sub-Saharan Africa have indicated that mortality reporting within health-care facilities is often incomplete, requiring follow-up tracing for greater accuracy [47]. Thus, the true rates of mortality in both cohorts were likely higher than those reported, based on facilities’ vital registry data [48]. Additionally, in measuring retention in care, Chan et al. did not account for possible transfers to other facilities when patients were considered lost to follow-up (LTFU) [32]. By contrast, Scheibe et al. corrected rates of attrition for documented instances of patient transfers to different facilities [37].

Both studies were judged to have serious risk of bias, particularly in two domains. Baseline confounding due to biased allocation into study arms, without sufficient controls for baseline differences, was a source of bias for both studies. Additionally, the Ugandan study had serious risk of selection bias for participation in the study, due to excluding potential participants who did not have patient files (16.9% of the sample, across all ages) [37]. The study in Malawi also faced serious risk of bias from delivering the integrated primary care co-intervention, the effects of which could not be separated from those of decentralising ART care [32]. Alongside decentralising HIV care, Chan et al. were assessing the effects of integrating primary care into HIV care at lower-level facilities, with provision of physical resources and staff training to implement this integrated care model. Full details of authors’ judgment for risk of bias for each domain are available in Additional File 3.

### Study findings

Table 2 summarises the characteristics and main findings of included studies, restricted to adolescent- and youth-specific data. For comparison between decentralised and centralised arms, neither study reported youths’ outcomes beyond attrition-related data. While Chan et al. presented retention and care and mortality outcomes separately, Scheibe et al. reported on overall attrition, which is comprised of both loss to follow-up and mortality [37,38]. Below, we present findings from both studies on these outcomes for adolescents and youths aged 10–24 at baseline.

**Table 2.** Included studies’ characteristics and main findings based on age-disaggregated data (10–24 years old).

Study author(s) and year of publication	Study design	Study location and dates	Adolescent and youth sample size	Adolescent and youth characteristics	Outcomes measured	Main results
Scheibe et al., 2013 [37]	Retrospective cohort study	Iganga District, Uganda, April 2004-September 2009	n = 63 (decentralised n = 16)	Gender: 71.4% female Age (median, IQR): 19 (13–22)	Attrition rate Probability of retention on ART	In centralised arm, 55.3% (n = 26) dropped out; in the decentralised arm, 37.5% dropped out (n = 6) Overall probability of retention on ART for youths 10–24 was 0.71 (95%CI 0.58–0.80) at 6 months, 0.62 (95%CI 0.48–0.73) at 12 months, and 0.45 (95%CI 0.31–0.58) at 18 and 24 months Receiving centralised care was not significantly associated with attrition from care in multivariate Cox analysis (aHR 1.26 [95%CI 0.42–3.81], *p*= 0.681)
Chan et al., 2010 [32]	Retrospective cohort study	Zomba District, Malawi, October 2004-December 2008	n = 1062 (decentralised n = 436)	Gender: 75.1% female Age at initiation (median, IQR): 21 (14–23) in centralised, 21 (14–23) in decentralised	WHO stage at ART initiation (n) Mortality (n) Defaulted (n)	Using crude ORs, there was no significant difference between study arms for WHO Stage I/II vs. Stage III/IV at initiation (OR 0.92 [95%CI 0.70–1.22], *p*= 0.585) Using crude ORs, decentralised care was significantly associated with a lower rate of mortality (OR 0.14 [95%CI 0.07–0.29], *p*< 0.001) Using crude ORs, decentralised care was significantly associated with a lower rate of defaulting from care (OR 0.37 [95%CI 0.26–0.55], *p*< 0.001)

aHR: Adjusted hazard ratio; CI: Confidence interval; IQR: Interquartile range; OR: Odds ratio; WHO: World Health Organisation.

In Uganda, a total of 63 participants included in the study were aged 10–24 at baseline, among whom the median age was 19 years (IQR 13–22) [37]. Decentralisation status was determined by the site of ART initiation, with 47 (74.6%) initiating at the hospital and 16 (25.4%) initiating at a health centre. Because ART was available at the hospital before the health centres, the median time on ART was longer for centralised adolescents and youths (p < 0.001). Attrition from care was defined as not attending the facility at least once during the 90 days prior to the audit in September 2009 (including mortality), except for documented transfers to another facility. In the centralised arm, attrition from care was observed for 26/47 (55.3%) youths, of whom 13 (50%) had passed away and 8 (30.8%) had been officially classified as lost to follow-up. By contrast, in the decentralised arm, 6/16 (37.5%) demonstrated attrition from care according to the study definition. However, in a multivariate Cox regression, decentralised care did not significantly predict attrition from care (aHR 0.79 [95%CI 0.26–2.40], *p*= 0.681), controlling for sex, most recent WHO stage, and time from HIV test to ART start. Only 56 participants were included in this analysis, because complete-case analysis was applied and 7 participants were missing at least one datapoint included in the regression.

In Malawi, among the 1062 participants aged 10–24 at baseline in the study, median age at initiation was 21 (IQR 14–23) [32]. Decentralised patients were designated as those receiving ART management at a health centre rather than the central hospital (n = 436, 41.1%). Study authors provided data on the total number of participants in each arm who reached the final outcomes of all-cause mortality and defaulting from care. Defaulting was defined as not attending the facility for >3 months since the last scheduled visit, censored to the end of data collection. Testing for uncontrolled between-group differences via Chi-square test, decentralised participants were significantly less likely than centralised participants to have passed away (OR 0.14 [95%CI 0.07–0.29], *p*< 0.001) and less likely to have defaulted from care (OR 0.37 [95%CI 0.26–0.55], *p*< 0.001). However, because individual-level data were not available for the 10–24-year-old subsample, we were not able to calculate adjusted odds ratios for outcomes, accounting for other covariates such as gender, WHO stage at initiation, or duration on ART.

## Discussion

Although decentralised ART has been rolled out across LMICs, we identified only two studies that reviewed its effectiveness for adolescent and youth health outcomes. Both were retrospective cohort studies from sub-Saharan Africa that analysed clinic-based records for all ART patients at those facilities, across all ages.

After disaggregating data for participants aged 10–24, findings in both studies were limited to attrition-related outcomes, such as retention in care and mortality. The heterogeneity in implementation of decentralisation and limited availability of age-specific data between the two studies did not allow for meta-analysis. Furthermore, the studies approximated attrition using non-interchangeable definitions. While the study in Uganda evaluated overall attrition (inclusive of all-cause mortality but adjusting for facility transfers), the study in Malawi defined defaulting from care separate from all-cause mortality (without adjusting for facility transfers). Because both studies relied solely on retrospective review of facilities’ vital registries to determine mortality, both likely underestimate the amount of attrition due to mortality [48]. By accounting for transfers, Scheibe et al. provided a more conservative and realistic estimate of true attrition from care in Uganda, while Chan et al. likely overestimated attrition by not accounting for transfers in Malawi.

Nevertheless, results from these two studies indicated the potential for decentralised HIV care to result in at least equivalent attrition-related outcomes as routine, centralised care for adolescents and youth. Both studies reported low rates of overall retention in HIV care for this population – consistent with findings from previous studies – but decentralising ART delivery seemed to provide a sustainable model for servicing a growing patient population in resource-limited settings [49]. However, to maintain safe and efficacious delivery of care, the actual implementation of decentralisation is critical.

In achieving at least equivalent outcomes on decentralised care, Chan et al. particularly note the significance of differentiating care across primary versus tertiary sites based on patient stability and care needs [38]. In theory, decentralisation should allow for patient mobility across care levels, such that patients presenting with complications at a primary clinic, including new opportunistic infections or treatment failure, should be able to return to tertiary care until reaching stability again [26]. Hence, these studies confirm the importance of proper selection of patients suitable for decentralised care at primary sites.

Furthermore, both studies highlight the need to consider service and supply factors within decentralised care, beyond the relocation of stable patients to primary care sites. In particular, both studies note that differences in performance between decentralised and centralised sites may be more dependent on clinical staff-related factors such as staff retention and turnover as well as supply-side factors like consistent availability of antiretroviral drugs and laboratory services [37,38].

However, the quality and sample sizes of data do not permit clear conclusions to be drawn for this population in LMICs, particularly as both studies did not sufficiently match the control groups or account for potential biases.

The study in Uganda reported that decentralised patients were significantly less likely to die or drop out of care. However, the application of complete case analysis excluded potential participants without patient files. Because hospital-based patients were found to be more likely to lack patient files, this mode of analysis introduces potential survivorship bias, likely towards null or favouring decentralised outcomes [37]. Additionally, analyses did not sufficiently adjust for baseline differences between decentralised and centralised groups. The higher morbidity and longer duration in care within the centralised arm could also have biased results towards null. Similarly, Chan et al. noted that there was significant selection bias in allocation to study arms, whereby stable patients were more likely to receive decentralised care. This selection bias introduces bias towards positive outcomes for decentralised youths. Because of the limited data available for adolescents and young people specifically, between-group comparisons for rates of mortality and retention in care could not be adjusted for baseline clinical staging or other covariates, as was done in the full sample.

Indeed, most studies on the effectiveness of decentralised care in paediatric and adult cohorts are limited by similar challenges in between-group controls [50–52]. Some studies controlled for differences in baseline health profiles of study arms by adjusting for CD4 values, but adjustments were limited by incomplete coverage of CD4 testing, often disproportionately in the decentralised arm [22,42,53]. However, one of the more rigorously designed cohort studies – which controlled for baseline BMI, history of ART use, CD4 count, and WHO stage – found higher mortality and attrition among centralised adults on ART in rural Malawi, compared to decentralised adults [39].

Generalizability of findings from included studies is further limited by the narrow feasibility and applicability of the decentralisation model tested in Malawi, given the intensive, frequent NGO support [37]. In the early years of decentralisation rollout, including the years of data collection, substantial NGO support was frequent in order to build capacity and test feasibility for this new model of care [54–56]. However, in recent years, such intensive NGO support has become increasingly uncommon in public facilities and varies across and within countries, making interventions run with substantial NGO support non-representative [57]. Since 2010, although the number of patients receiving decentralised HIV care has increased, responsibility for delivering sustainable decentralised care has significantly shifted towards public facilities themselves [58,59].

Nevertheless, this review identifies an important gap in knowledge about the effects of this widespread public health approach for this particularly vulnerable population, which has already been rolled out in many LMICs. Several cohort studies trace the health outcomes of adolescents and young persons receiving decentralised care. However, this review found only two that allowed for the comparison of their health outcomes while in decentralised versus centralised care, which would inform how best to optimise care for this population [13,14,17,18,60]. The literature gap identified here has several key implications for future research and practice.

Although 11 potentially eligible studies included adolescents and youths within their cohorts, only 2 studies were able to disaggregate data for them. In part, the limited data available for this population is because the WHO and many other organisations have summarised adult and paediatric data as ≥15 years and <15 years categories, respectively [9]. Consequently, national data specific to health outcomes for this population are frequently unavailable. Due to recognition of the importance of this vulnerable population in recent years, countries have been requested to disaggregate data for this population. However, most countries lack sufficient health system infrastructure to do so. This review contributes to the literature indicating the urgent need for strengthened health systems that allow for this more granular focus on adolescents and youths within routine health monitoring programming [61,62].

Furthermore, this review highlights another key gap in the literature: the scarcity of studies evaluating the effects of decentralised HIV care within the past 5–8 years. More recent studies would reflect the current reality of decentralisation, rather than the landscape in its early years. In fact, of the 11 studies whose authors were contacted for age-disaggregated data, only 4 had any post-2011 data [43–46]. Thus, this review indicates the urgent need for more up-to-date studies evaluating decentralised HIV care as it is currently being implemented, using recent – and therefore relevant – health outcome data.

Finally, across studies evaluated for this review, outcome data were most comprehensive for attrition-related measures, including mortality and loss to follow-up. Very few studies of decentralisation, across all ages, evaluated health outcomes beyond ‘end-of-care’ events, such as virological, immunological, or morbidity outcomes. Only 3 of the 11 potentially eligible studies for this review included any outcomes beyond mortality, loss to follow-up, and transfers out of care [39,43,46]. This review highlights the need for studies to rigorously evaluate the effects of decentralisation on these non-terminal outcomes, which provide a more comprehensive understanding of the lived experience of ART patients and their outcomes while within care.

This review provides a comprehensive assessment of the effect of decentralising HIV care for adolescents and youths living in LMICs. It is the first systematic review to focus on this model of care delivery for this population. Strengths of this review include its inclusive search strategy, including grey literature and multiple databases and allowing for the age disaggregation of data for studies including the target population. Additionally, this review provided rigorous critical appraisal of included studies’ quality and risk of bias. However, limitations include restriction of the search to English-language studies, which may have omitted evaluations published in other languages. An additional limitation is suboptimal reporting of outcome data for adolescents and youths in the included studies, which did not allow for meta-analysis of findings and subsequently for clear conclusions to be drawn.

## Conclusions

As decentralised ART care continues to scale up globally, further primary research is urgently required to evaluate the effects of decentralising HIV care delivery for adolescents and young people in LMICs, particularly using recent data that reflects the current landscape of health systems’ care burdens. Although decentralised ART delivery is already being implemented worldwide, further research is required to evaluate the efficacy of this approach for HIV-positive youth in order to identify opportunities for optimising care outcomes. Importantly, more rigorous study designs are required to truly examine the effect of decentralisation, as well as evaluations of outcomes beyond attrition that characterise health progression within care. Existing evidence is limited in both quantity and quality. The evidence base on decentralisation is largely limited to adult and paediatric populations, with very few studies able to provide adolescent- and youth-specific data that would allow for an understanding of how this mode of care delivery affects one of the most vulnerable populations living with HIV.

## Paper context

To scale up care, resource-limited countries are decentralising HIV care delivery. Youth demonstrate the worst HIV outcomes of all age groups. This systematic review evaluates the effect of decentralising HIV care on youth health outcomes in low- and middle-income countries. Two studies met inclusion criteria. Both were out-dated and poorly controlled, limiting implications for the present context. This review highlights the urgent need to better understand how decentralising HIV care may affect this vulnerable population.
